# Comparative Analysis of the Sedative and Hypnotic Effects among Various Parts of *Zizyphus spinosus* Hu and Their Chemical Analysis

**DOI:** 10.3390/ph17040413

**Published:** 2024-03-25

**Authors:** Baojian Li, Yuangui Yang, Zhongxing Song, Zhishu Tang

**Affiliations:** 1 Shaanxi Collaborative Innovation Center of Chinese Medicine Resources Industrialization, State Key Laboratory of Research & Development of Characteristic Qin Medicine Resources (Cultivation), Shaanxi Innovative Drug Research Center and College of Pharmacy, Shaanxi University of Chinese Medicine, Xianyang 712046, China; lbj17690949096@163.com (B.L.); szx74816@sina.com (Z.S.); 2 China Academy of Chinese Medical Sciences, Beijing 100700, China

**Keywords:** *Zizyphus spinosus*, various parts, sedative and hypnotic effect, chemical analysis

## Abstract

*Zizyphus spinosus* Hu (ZS), as a “medicinal and food-homologous” plant, has been used for a long history. The study was to assess the sedative and hypnotic effects among various parts of ZS. The model, diazepam (DZP), ZS kernel (ZSS), ZS flesh (ZSF), and ZS husk (ZSKS) group occurred subsequent to the successful establishment of the para-chlorophenylalanine induced insomnia model via intraperitoneal injection. The latency and duration of sleep in mice in each group were recorded. The substance basis of various parts of ZS was analyzed by the UPLC-QTOF-MS technique. The results showed that relative to the model group, DZP, ZSS, ZSF, and ZSKS groups demonstrated shortened sleep latency (*p* < 0.05) and extended sleep duration (*p* < 0.01). The GABA, 5-HT, and BDNF levels were significantly upregulated in the brain tissues of the mice in the DZP, ZSF, and ZSS groups (*p* < 0.01). However, the improvement in ZSKS was non-significant. Additionally, the mRNA and protein expression levels of 5-HT1AR, GABAARα1, and BDNF in mice in the DZP, ZSS, and ZSF groups were significantly enhanced (*p* < 0.01). However, the improvement in the ZSKS group was insignificant (*p* < 0.05). The examination of the substance composition across different parts revealed that the shared chemical basis contributing to the sedative and hypnotic potency of different parts of ZS may involve the presence of compounds such as (**1**) magnoflorine, (**8**) betulinic acid, (**9**) ceanothic acid, and (**10**) alphitolic acid. It provides a basis for further elucidation of the substance basis responsible for the functional and medicinal effects of ZS.

## 1. Introduction

Insomnia stands as one of the prevalent disorders in contemporary clinical practice, with its incidence steadily rising each year. Presently, approximately 30% of the global population experiences at least one symptom of insomnia, exerting a noteworthy impact on their daily lives. Insomnia manifests as challenges in falling asleep, maintaining sleep, or achieving restorative sleep. This condition is accompanied by notable symptoms, including fatigue, decreased concentration, impaired cognitive function, irritability, anxiety, and depressed mood [1,2,3,4,5]. The onset of insomnia could be associated with certain neurotransmitters, like γ-aminobutyric acid (GABA), 5-hydroxytryptamine (5-HT), and brain-derived neurotrophic factor (BDNF). The levels of these neurotransmitters were found to be significantly reduced in the brains of insomniac mice [6,7,8]. 5-HT, recognized as the “pleasure neurotransmitter”, exerts its functions within the brain through its extensive family of receptors. Notably, the 5-HT1 subfamily has emerged as a crucial target for therapeutic medications aimed at addressing psychiatric disorders [9,10,11]. GABA serves as a critical inhibitory neurotransmitter within the central nervous system (CNS), with the GABA A-type receptor (GABAAR) being mainly associated with insomnia symptoms [12,13,14]. Additionally, as a significant molecular component of neuroplasticity, BDNF can enhance sleep by enhancing synaptic plasticity and promoting neurogenesis, particularly within the hippocampus [15,16,17].

Currently, medications utilized for managing insomnia primarily encompass benzodiazepine receptor agonists, melatonin and its receptor agonists, antidepressants, antihistamines, and various other compounds. Despite their considerable efficacy in alleviating insomnia symptoms, many of these drugs are addictive and categorized as controlled substances, consequently constraining their usage [18,19,20,21]. Traditional Chinese medicine (TCM) therapy presents significant advantages over chemical drugs, including favorable safety profiles, non-addictive properties, affordability, and ease of accessibility. However, there is a scarcity of current research investigating the mechanisms of action associated with TCM therapy and the underlying substance basis for its pharmacological effects [22,23,24]. In TCM, various herbs are employed for the treatment of insomnia, encompassing both compound preparations and individual herbs, such as Zao Ren An Shen Capsules and Sour Zao Ren [25,26,27].

*Zizyphus spinosus* Hu (ZS) is a woody plant belonging to the Rhamnaceae family, as a “medicinal and food-homologous” plant, primarily found in regions across China, including Shaanxi, Shanxi, Hebei, and Shandong. Its annual production exceeds 30,000 tons. The Herbal Classic of Shen Nong recorded that “ZS has a sour and flat flavor and is distributed in rivers and pools. ZS can treat cold and heat in the heart and abdomen, pathogenic factor gathering, limb pain, and wet paralysis. Taking ZS for a long time can consolidate the five internal organs, improve health status, and prolong life”. In the literature from the Sui, Tang, and Song dynasties, there are documented records of ZS kernel (ZSS) being utilized for medicinal purposes. For instance, Ben Jing recorded that ZSS possesses therapeutic properties capable of addressing insomnia, fortifying the liver, and nourishing qi. These descriptions highlight the historical recognition of the medicinal functions attributed to ZSS during that time. In the Synopsis of Golden Chamber, it is reported that insomnia resulting from deficiency may find relief through the consumption of ZS soup. The term “ZS Soup” rather than “ZSS”, implies that both ZS and ZSS may offer similar medicinal benefits. However, at present, ZSS is predominantly employed as the traditional medicinal component. At the same time, other parts, such as ZS husk (ZSKS) and ZS flesh (ZSF), are often discarded, leading to resource wastage and ecological environmental destruction. The Chinese Pharmacopoeia recognizes ZSS for its sedative and hypnotic effects. ZSS primarily comprises flavonoids, saponins, alkaloids, fatty acids, and other components. These components exhibit diverse biological activities, including sedative and hypnotic effects, antioxidant properties, anti-inflammatory actions, and cardioprotective benefits [28,29]. ZSF contains flavonoids, polysaccharides, alkaloids, and other components, which demonstrate biological activities such as enhancing sleep, protecting the liver, and exhibiting anti-tumor effects [30]. However, there is limited research on ZSKS, and the substance basis underlying the varied medicinal effects of different parts of ZS has rarely been reported.

The objective of the present research was to assess the efficacy of ZSS, ZSF, and ZSKS in the PCPA-induced insomnia model in mice. Additionally, the chemical composition of various parts of ZS was investigated by UPLC-MS/MS to analyze the pharmacological basis for the sedative-hypnotic effect of different parts of ZS. The outcomes of this investigation offer a theoretical groundwork for comprehensive utilization of the non-medicinal components of ZS.

## 2. Results and Discussion

### 2.1. Pharmacodynamic Evaluation Results of Different Parts of ZS

#### 2.1.1. Sleep Latency and Sleep Duration

**Figure 1:**
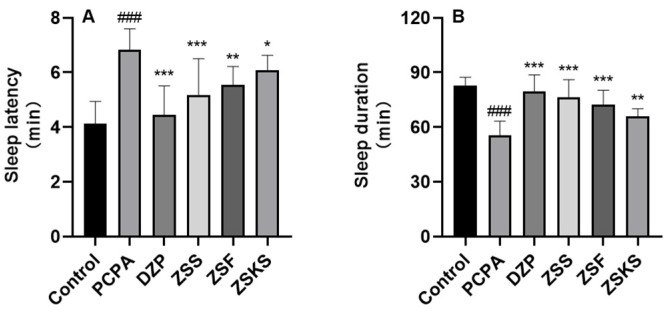
Effects of various parts of ZS on sleep latency and duration in PCPA-induced insomnia model mice. Note: (**A**) Sleep latency; (**B**) Sleep duration. Note: Relative to the blank group, ### represents *p* < 0.001; in comparison to the model group, *** reflects *p* < 0.001, ** reflects *p* < 0.01, and * reflects *p* < 0.05.

As depicted in Figure 1A, the model group exhibited a considerable increase in sleep latency in comparison to the blank group (*p* < 0.001). Both the DZP and various parts groups demonstrated a reduction in sleep latency in comparison to the model group (*p* < 0.05). The ZSS group demonstrated the shortest sleep latency, followed by the ZSF and ZSKS groups. As depicted in Figure 1B, a noteworthy reduction in sleep duration was observed in the model group in comparison to the blank group (*p* < 0.001). However, an extension in sleep duration was noted in both the DZP and various parts groups in comparison to the model group (*p* < 0.01). Notably, the ZSS group exhibited the longest sustained sleep duration, followed by the ZSF and ZSKS groups.

#### 2.1.2. HE Staining

**Figure 2:**
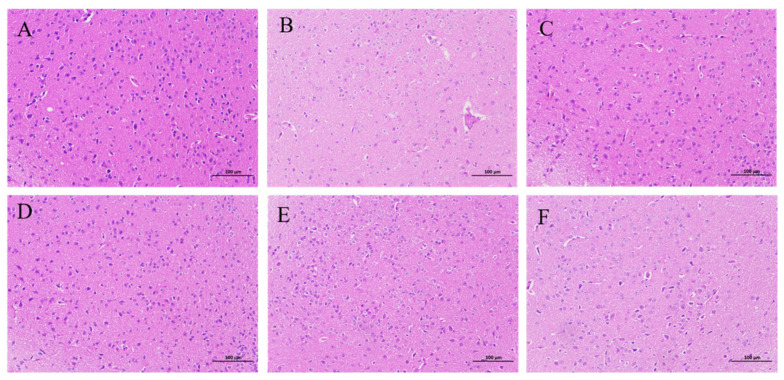
Effects of various parts of ZS on the histopathological morphology of mice brain tissue (HE, ×200). Note: (**A**) Blank group; (**B**) Model group; (**C**) DZP group; (**D**) ZSS group; (**E**) ZSF group; (**F**) ZSKS group.

HE staining (Figure 2) revealed irregularities in cellular arrangement within the brain tissue of the model group relative to the blank group. Specifically, the hypothalamus demonstrated numerous wrinkled and deeply stained neuronal nuclei alongside shrunken and deformed cell bodies, as well as poorly defined boundaries between nuclei and cytoplasm. Furthermore, a significant portion of neurons in the hypothalamus exhibited signs of degeneration, accompanied by sparse cytoplasmic staining. In comparison to the model group, clear staining patterns were evident in both the DZP and various parts groups. A small number of neurons in the hypothalamus area had improved nuclear wrinkling, as well as the degeneration of a small number of neurons. These observations imply that distinct components of ZS enhanced the cytomorphology of mouse brain tissue.

#### 2.1.3. ELISA Analysis

The examination of GABA, 5-HT, and BDNF content in brain tissues is crucial for evaluating the amelioration of insomnia symptoms via various components of ZS [31,32]. These neurotransmitters are crucial in nerve signal transmission. Certain drugs with sedative and hypnotic effects typically regulate neurotransmitter levels to exert their pharmacological effects.

**Figure 3:**
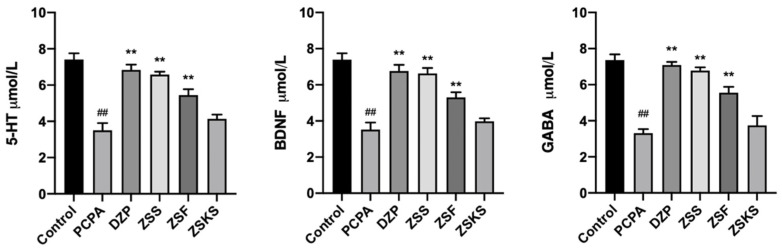
Measurement of GABA, 5-HT, and BDNF content in the brain tissue of mice in each group by ELISA. Note: Relative to the blank group, ## signifies *p* < 0.01; comparison to the model group, ** signifies *p* < 0.01.

The ELISA results (Figure 3) indicate that, in comparison to the blank group, the levels of 5-HT, GABA, and BDNF in the model group were significantly decreased (*p* < 0.01). Conversely, relative to the model group, the DZP and various parts groups exhibited significantly increased content of GABA, 5-HT, and BDNF (*p* < 0.05). Notably, the descending order of 5-HT, GABA, and BDNF content across different components of ZS was as follows: ZSS, ZSF, and ZSKS.

#### 2.1.4. PCR Analysis

**Figure 4:**
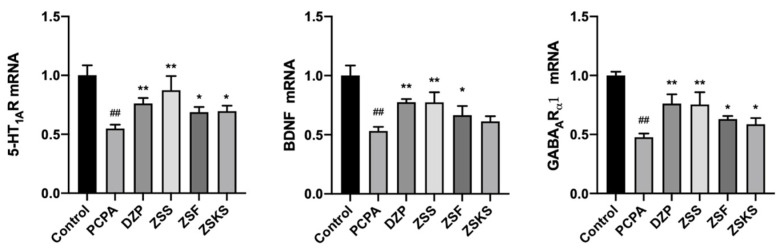
The mRNA expression levels of 5-HT1AR, GABAARα1, and BDNF in each group of mice measured by PCR. Note: In comparison to the blank group, ## signifies *p* < 0.01; comparison to the model group, ** signifies *p* < 0.01, * signifies *p* < 0.05.

The pathogenesis of insomnia is unclear, and several hypotheses exist [33]. Key neurotransmitters like 5-HT and GABA are closely linked to central neurological disorders, including insomnia and memory impairment. In individuals with insomnia, there is a significant decrease in levels of 5-HT and GABA neurotransmitters. Therefore, they serve as important indicators for assessing the severity of insomnia [34,35,36]. The function of 5-HT in the brain is closely related to its large family of receptors. In particular, the 5-HT1 subfamily is emerging as a significant target for therapeutic drugs in conditions such as insomnia [37]. GABA is an inhibitory neurotransmitter associated with CNS, whereas GABAAR is closely associated with symptoms of insomnia [38]. Expression levels of BDNF, a brain-derived neurotrophic factor, correlate with the trophic status of brain neurons and can be used to evaluate insomnia, memory impairment, and other CNS-related diseases [15]. The PCR results (Figure 4) indicated a significant reduction in the expression levels of mRNA of 5-HT1AR, GABAARα1, and BDNF in the model group in comparison to the blank group (*p* < 0.01). Relative to the model group, the mRNA expression levels of 5-HT1AR, GABAARα1, and BDNF were elevated in DZP, ZSS, and ZSF groups (*p* < 0.05). Additionally, the mRNA expression levels of 5-HT1AR and GABAARα1 were elevated in the ZSKS group (*p* < 0.05), although the elevation of BDNF mRNA expression was insignificant (*p* > 0.05). In different parts of ZS, the mRNA expression levels of 5-HT1AR exhibited the following descending order: ZSS, ZSKS, and ZSF. Similarly, the mRNA expression levels of GABAARα1 followed the descending order: ZSS, ZSF, and ZSKS. Additionally, the mRNA expression levels of BDNF showed the following descending order: ZSS, ZSF, and ZSKS.

#### 2.1.5. Western Blotting

**Figure 5:**
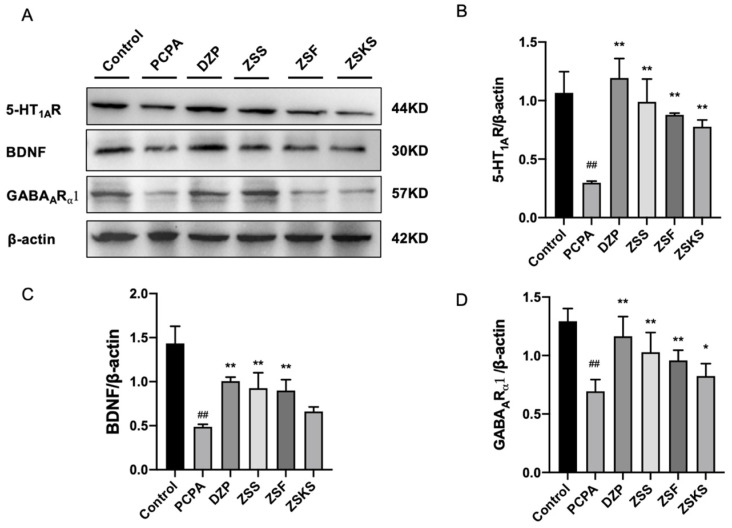
(**A**) Western blotting analysis of 5-HT1AR, GABAARα1, and BDNF in mice of each group; (**B**) Proportion of immunoblotting bands of 5-HT1AR, (**C**) GABAARα1, and (**D**) BDNF relative to Beta Actin. Note: In comparison to the blank group, ## represents *p* < 0.01; relative to the model group, ** represents *p* < 0.01, * represent *p* < 0.05.

Western blotting results, as depicted in Figure 5, revealed a significant downregulation in protein expression of 5-HT1AR, GABAARα1, and BDNF in the model group in comparison to the blank group (*p* < 0.01). Conversely, in comparison to the model group, both the DZP and various parts groups demonstrated a substantial elevation in protein expression of 5-HT1AR and GABAARα1 (*p* < 0.05). Similarly, DZP, ZSS, and ZSF demonstrated a significant elevation in the protein expression of BDNF (*p* < 0.01). Conversely, the impact of ZSKS was insignificant (*p* > 0.05). The protein expression levels of 5-HT1AR, GABAARα1, and BDNF in different parts of ZS exhibited the following descending order: ZSS > ZSF > ZSKS.

### 2.2. The Constituents Analysis of Different Parts of ZS

**Figure 6:**
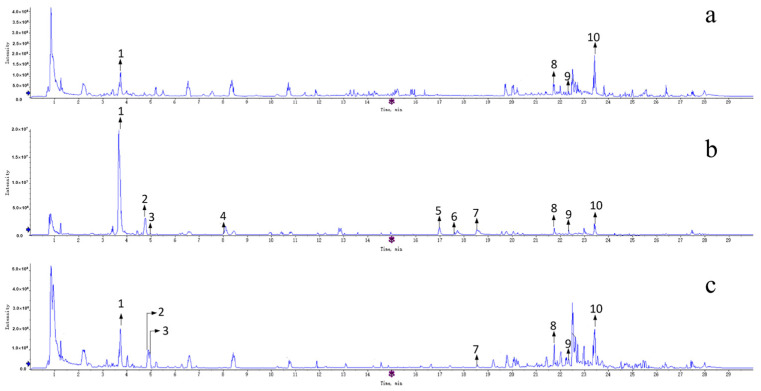
Total ions chromatograms of different parts of ZS. Notes: (**a**) ZSKS; (**b**) ZSS; (**c**) ZSF; 1. Magnoflorine; 2. Spinosin; 3. Rutin; 4. 6‴-Feruloylspinosin; 5. Jujuboside A1; 6. Jujuboside A; 7. Jujuboside B; 8. Betulinic acid; 9. Ceanothic acid; 10. Alphitolic acid.

**Figure 7:**
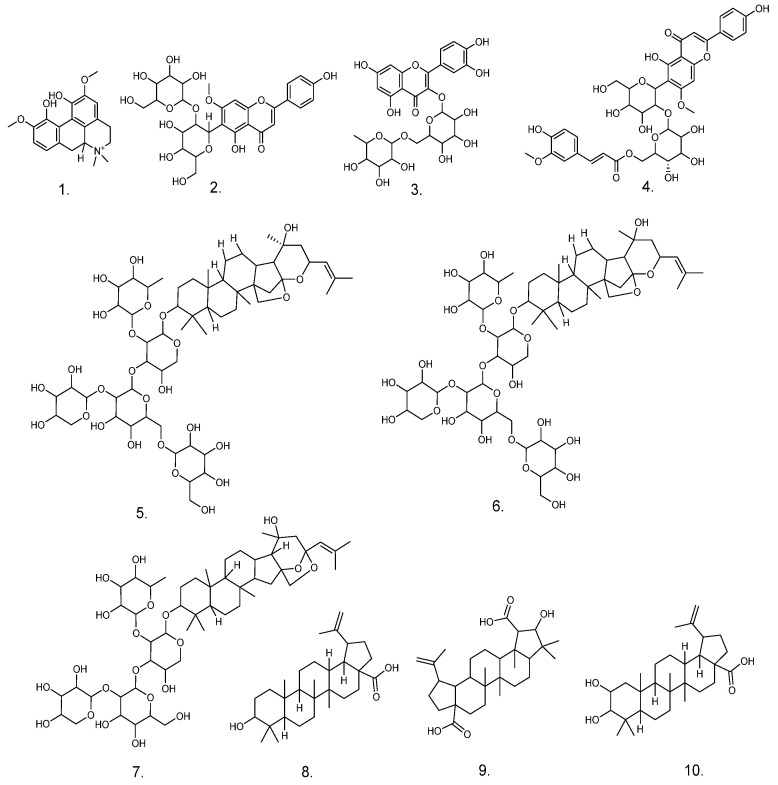
Compounds identified in the Zizyphus spinosus extracts. Notes: 1. Magnoflorine; 2. Spinosin; 3. Rutin; 4. 6‴-Feruloylspinosin; 5. Jujuboside A1; 6. Jujuboside A; 7. Jujuboside B; 8. Betulinic acid; 9. Ceanothic acid; 10. Alphitolic acid.

The total ion chromatograms of UPLC-QTOF-MS/MS for various parts of ZS in positive ion mode are illustrated in Figure 6. The chemical constituents of different parts of ZS exhibited robust responses in positive ion mode, with quasi-molecular ion peaks predominantly observed as [M+H]^+^. Based on the relevant literature data and the mass spectral information of some controls, a comprehensive analysis identified and postulated the presence of ten chemical components across various parts of ZS. These components comprise one alkaloid, three flavonoids, three saponins, and three triterpenoids (Table 1). The mass spectrometry data of compounds in ZSS, as separated by UPLC, are delineated in Table 1. The structural formulas of compounds are depicted in Figure 7.

#### 2.2.1. Alkaloid

In Table 1, magnoflorine (compound No. **1**) exhibited a quasi-molecular ion peak of *m/z* 342.1673 [M+H]^+^ in the positive mode, with a presumed chemical formula of C_20_H_24_NO_4_. Subsequently, secondary mass spectrometry (SMS) analysis was performed using *m/z* 342.1673 [M+H]^+^ as the parent ion. The resulting secondary mass spectra showed distinct fragment ions *m/z* 297, *m/z* 282, *m/z* 265, *m/z* 237, and *m/z* 222. Comparison with a prior reference confirmed the identity of the compound as magnoflorine [39].

#### 2.2.2. Triterpenoid

As shown in Table 1, the mass spectrum of ceanothic acid (compound No. **9**) yielded the quasi-molecular ion peak *m/z* 487.3376 [M+H]^+^ in the positive ion [M+H]^+^ mode with a presumed chemical formula of C_30_H_46_O_5_. SMS was carried out using *m/z* 487.3376 [M+H]^+^ as the parent ion. The resulting secondary mass spectra exhibited distinct fragment ions at *m/z* 459, *m/z* 312, *m/z* 235, and *m/z* 190. Per the literature data, this compound was proposed to be ceanothic acid [40].

The mass spectrum of betulinic acid (compound No. **8**) revealed a quasi-molecular ion peak *m/z* 457.1672 [M+H]^+^ in the positive [M+H]^+^ mode with a presumed chemical formula of C_30_H_48_O_3_ as shown in Table 1. SMS analysis was carried out using *m/z* 457.1672 [M+H]^+^ as the parent ion. The resulting secondary mass spectra showed distinct fragment ions *m/z* 439, *m/z* 411, *m/z* 281, *m/z* 248, *m/z* 202, and *m/z* 119. As per the loss of neutrality and the assessed literature data, this compound was proposed to be betulinic acid [40].

As depicted in Table 1, the mass spectrum of maltogenic acid (compound no. **10**) produced the quasi-molecular ion peak *m/z* 495.3442 [M+Na]^+^ in the positive [M+Na]^+^ mode, with a presumed chemical formula of C_30_H_48_O_4_. SMS analysis was carried out utilizing *m/z* 495.3442 [M+Na]^+^ as the parent ion. The obtained secondary mass spectra showed distinct fragment ions *m/z* 437, *m/z* 338, *m/z* 301, *m/z* 281, *m/z* 207, and *m/z* 149. According to the literature data, this compound was hypothesized to be alphitolic acid [40].

#### 2.2.3. Flavonoid

As indicated in Table 1, spinosin generated a quasi-ionic peak of *m/z* 609.1777 [M+H]^+^ in the positive [M+H]^+^ mode with a presumed chemical formula of C_28_H_32_O_15_. SMS analysis was carried out employing *m/z* 609.1777 [M+H]^+^ as the parent ion. The resulting secondary mass spectra exhibited distinct fragment ions *m/z* 489 and *m/z* 429. Further analysis with *m/z* 429 as the parent ion yielded *m/z* 351, *m/z* 327, and *m/z* 297 fragment ions. Comparison with previous references confirmed the composition as spinosin [39,41].

6‴-feruloylspinosin yielded a quasi-ionic peak at *m/z* 785.2250 [M+H]^+^ in the positive ion mode, with a presumed chemical formula of C_38_H_40_O_18_. SMS analysis was carried out utilizing *m/z* 785.2250 [M+H]^+^ as the parent ion. The resulting secondary mass spectra showed distinct fragment ions *m/z* 665, *m/z* 609, *m/z* 429, *m/z* 351, *m/z* 327, and *m/z* 177. Comparison with previous references confirmed the composition as 6‴-feruloylspinosin [39,41]. 

As depicted in Table 1, in the positive [M+Na]^+^ mode, rutin (compound no. **3**) yielded a quasi-ionic peak of *m/z* 633.1382 [M+Na]^+^ with a presumed chemical formula of C_27_H_30_O_16_. SMS analysis was conducted utilizing *m/z* 633.1382 [M+Na]^+^ as the parent ion. The resulting secondary mass spectra exhibited distinct fragment ions *m/z* 465, *m/z* 303, and *m/z* 229. These fragment ions were consistent with the characteristic fragmentation pattern observed when the parent ion of rutin in the control sample underwent cleavage. Further, confirmation of the composition as rutin was obtained through retention time and comparison with reference standards.

#### 2.2.4. Saponin

As illustrated in Table 1, in the positive [M+Na]^2+^ mode, Jujuboside A1 (compound No. **5**) produced a quasi-ionic peak of *m/z* 626.2757 [M+Na]^2+^ with a presumed chemical formula of C_58_H_94_O_26_. Subsequently, SMS analysis was carried out utilizing *m/z* 1206.5714 [M+H]^+^ as the parent ion. The fragment ion *m/z* 1075 resulted from the removal of one molecule of xylose from the parent ion. Subsequently, two molecules of glucose, one molecule of rhamnose, and one molecule of arabinose were consecutively lost, resulting in the formation of fragment ions *m/z* 913, *m/z* 751, *m/z* 605, and *m/z* 455. Additionally, *m/z* 1057 was the fragment ion formed by the loss of one molecule of xylose following the loss of one molecule of water from the parent ion. Based on literature data, this compound was hypothesized to be Jujuboside A1 [41].

**Figure 8:**
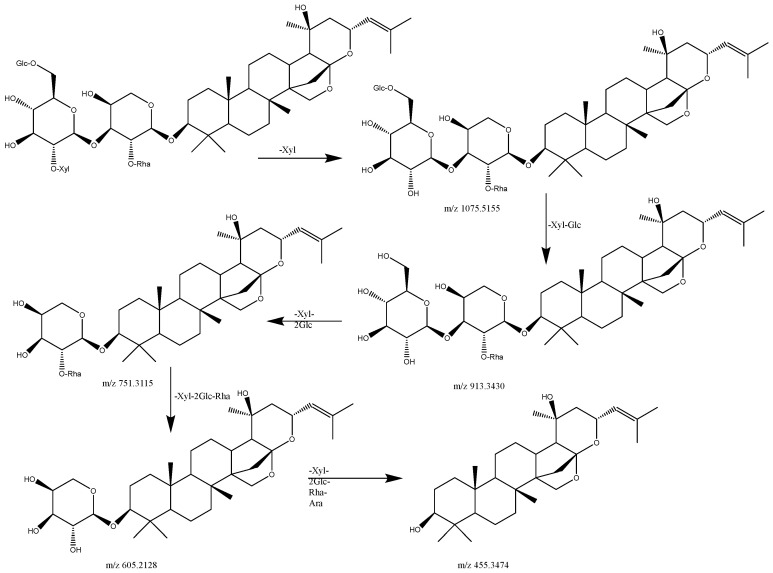
Possible fragmentation mechanism of Jujuboside A.

As shown in Table 1, Figure 8, in the positive [M+Na]^2+^ mode, Jujuboside A (compound No. **6**) generated a quasi-ionic peak of *m/z* 626.2857 [M+Na]^2+^ with a presumed chemical formula of C_58_H_94_O_26_. Additionally, SMS analysis was carried out with *m/z* 1206.5714 [M+H]^+^ as the parent ion. The fragment ion at *m/z* 1075 resulted from the removal of one molecule of xylose from the parent ion. Following this, two molecules of glucose, one molecule of rhamnose, and one molecule of arabinose were successively lost, giving rise to the fragment ions at *m/z* 913, *m/z* 751, *m/z* 605, and *m/z* 455. Moreover, the fragment ion at *m/z* 1057 was generated by the loss of one molecule of xylose following the loss of one molecule of water from the parent ion. According to literature data, this compound was proposed to be Jujuboside A [39,42].

As indicated in Table 1, in the positive [M+Na]^2+^ mode, Jujuboside B (Compound No. **7**) produced a quasi-ionic peak of *m/z* 545.2591 [M+Na]^2+^ with a presumed molecular formula of C_52_H_84_O_21_. Subsequently, SMS analysis was carried out with *m/z* 1044.5902 [M+H]^+^ as the parent ion. *m/z* 913 was the fragment ion resulting from the parent ion removing one molecule of xylose. Then, the loss of one molecule of rhamnose and one molecule of glucose formed the fragment ion *m/z* 605. *m/z* 895 and *m/z* 751 were fragment ions formed by the loss of one molecule of water from the parent ion and the removal of one molecule of xylose and 1 molecule of rhamnose. According to literature data, this compound was proposed to be Jujuboside B [39,41].

The examination of the substance composition across different parts revealed that the sour date fruit kernel (ZSS group) comprised the following compounds: (**1**) magnoflorine, (**2**) spinosin, (**3**) rutin, (**4**) 6‴-feruloylspinosin, (**5**) Jujuboside A1, (**6**) Jujuboside A, (**7**) Jujuboside B, (**8**) betulinic acid, (**9**) ceanothic acid, and (**10**) alphitolic acid. ZSF contained compounds (**1**), (**2**), (**3**), and (**7**–**10**), while ZSKS contained compounds (**1**) and (**8**–**10**). The common substance basis for the sedative and hypnotic effects of various parts of ZS may be the presence of compounds (**1**), (**8**), (**9**), and (**10**) across all parts of ZS. The substance basis for the higher efficacy of ZSS than ZSF and ZSKS may be due to the presence of compounds (**4**), (**5**), and (**6**) in ZSS, which are not present in ZSF and ZSKS. In addition, the substance basis for the higher efficacy of ZSF than ZSKS may be the presence of compounds (**2**), (**3**), (**7**), and (**8**) in ZSF, which are not present in ZSKS.

Studies have shown that Spinosin and Jujuboside A1 are the sedative bioactive constituents in Ziziphi Spinosae Semen (ZSS) and that these constituents contribute to the effects of improved learning and memory [43]. In addition, the antidepressant-like effects of Citrina extracts are mainly associated with the flavonoid flavins, especially rutin [44]. Cao J X et al. found that 4.3% of Jujuboside A and 5.0% of Jujuboside B possessed hypnotic activity as evidenced by the promotion of sleep in normal rats and enhancement of pentobarbital hypnotic activity in mice [45]. Furthermore, Fan L et al. identified 6‴-Feruloylspinosin as a Q-marker for the sedative and hypnotic effects of ZSS and Fried Ziziphi Spinosae Semen (FZSS) by LC-MS/MS and molecular docking analysis [46]. In our study, the efficacy of ZSS was better than that of ZSF and ZSKS, which could be attributed to the presence of sedative bioactive components such as 6‴-feruloylspinosin, Jujuboside A1, and Jujuboside A. The efficacy of ZSF was better than that of ZSKS, probably due to the presence of spinosin, rutin, and Jujuboside B in ZSF. This provides a basis for further elucidation of the pharmacological basis of ZS and for the development and exploitation of its resources. 

## 3. Materials and Methods

### 3.1. Materials and Reagents

ZS was sourced from Hengshan District, Yulin City, Shaanxi Province, and was identified as the dried fruit of ZS of Rhamnaceae. Song Zhongxing, Chief Pharmacist of the Provincial-Ministerial Collaborative Innovation Center for the Industrialization of Traditional Chinese Medicine Resources in Shaanxi Province, conducted this identification. In this research, PCPA (Sigma, Burlington, MA, USA), diazepam (Shandong Xinyi Pharmaceutical Co., Ltd., De Zhou City, China), and ELISA kits (Jiangsu Enzyme Immunity Industry Co., Ltd., Suzhou, China) for 5-HT, GABA, and BDNF were employed. Additionally, other reagents utilized included the control magnoflorine (A31HB193420, Shanghai yuanye Bio-Technology Co., Ltd., Shanghai, China), spinosin (111869-201203, National Institutes for Food and Drug Control, Beijing, China), rutin (HR1713S1, Baoji Herbest Bio-Tech Co., Ltd., Baoji, China), 6‴-feruloylspinosin (P04J125136476, Shanghai yuanye Bio-Technology Co., Ltd., Shanghai, China), Jujuboside A1 (A28HB181012, Shanghai yuanye Bio-Technology Co., Ltd., Shanghai, China), Jujuboside A (110734-201713, National Institutes for Food and Drug Control, Beijing, China), Jujuboside B (110814-201408, National Institutes for Food and Drug Control, Beijing, China), betulinic acid (111802-201001, National Institutes for Food and Drug Control, Beijing, China), ceanothic acid (HA061307, Baoji Herbest Bio-Tech Co., Ltd., Baoji, China), and alphitolic acid (HA060808, Baoji Herbest Bio-Tech Co., Ltd., Baoji, China). 

### 3.2. Preparation of Gavage Solution

#### 3.2.1. Preparation of PCPA Suspensions

In this procedure, 12 g of PCPA powder was combined with a 0.3% CMC-Na physiological saline solution, fixed to a volume of 300 mL, and thoroughly stirred to create a suspension. The resulting suspension had a mass concentration of 40 g/L.

#### 3.2.2. Preparation of Gavage Liquid from Different Parts of ZS

Herein, 100 g of ZSS, ZSF, and ZSKS underwent drying, powdering, and extraction through heating and refluxing with 70% ethanol. The resulting extracts were then freeze-dried to obtain lyophilized powder. The extraction rates of lyophilized powder for ZSS, ZSF, and ZSKS were 55.23%, 24.78%, and 35.66%, respectively. Subsequently, the lyophilized powder was mixed with purified water, and the volume was maintained at 100 mL. The mixture was stirred until dissolved, resulting in the formation of gavage solutions of the different parts of ZS, with a concentration of 1 g/mL of raw drug.

#### 3.2.3. Preparation of Diazepam Gavage Solution

Eight diazepam tablets, each containing 20 mg of diazepam, were crushed into powder. The powder was then mixed with pure water, resulting in a 100 mL gavage solution with 0.1 mg/mL concentration of diazepam.

### 3.3. Animals

Sixty female Kunming (KM) mice of specific pathogen-free (SPF) grade, weighing 40 ± 2 g, were procured from Chengdu Dashuo Laboratory Animal Co., Ltd., Chengdu, China, with Certificate of Conformity No. SCXK(Chuan)2020-030. The animals were accommodated in an environment with a room temperature maintained at 23 ± 1.5 °C and a relative humidity ranging between 50% and 60%. They were allowed ad libitum access to food and water, following a 12 h day/night cycle. The experimental procedure received approval from the Ethics Committee of Shaanxi University of Traditional Chinese Medicine, with the approval number SUCMDL20230227001.

### 3.4. Animal Modeling and Drug Administration

Sixty female KM mice were randomly stratified into six groups as follows, each comprising ten mice: (1) control group, (2) model group, (3) DZP group, (4) ZSS group, (5) ZSF group, and (6) ZSKS group. The mice were induced into the model state through intraperitoneal injection of PCPA. The PCPA suspension was administered intraperitoneally at a dose of 400 mg/kg daily for four consecutive days. With the exception of the blank group, all mice exhibited a progressive development of mania, biting behavior, heightened aggressiveness, disruption in circadian rhythm, withered hair, and reduced food intake over the 4 day modeling period. These observations collectively indicate the successful induction of the desired model. Subsequently, each group received daily gavage administration for eight consecutive days. The blank and model groups were administered pure water by gavage, while the remaining groups were administered the corresponding drugs orally at the following doses: DZP: 1.3 mg/kg; ZSS: 483.21 mg/kg; ZSF: 1077.44 mg/kg; ZSKS: 695.27 mg/kg. Upon completion of the sodium pentobarbital synergistic sleep experiment on the eighth day of administration, the mice were immediately euthanized, their heads were severed, and the entire brain tissue was extracted. A portion of the tissue was immersed in tissue fixative for future use, while the remaining portion was stored at −80 °C for subsequent analysis.

### 3.5. Sodium Pentobarbital Synergistic Sleep Experiment

Two hours following the administration on day eight, all groups of mice received an intraperitoneal injection of sodium pentobarbital at a suprathreshold dose of 100 mg/kg. Subsequently, sleep latency and sleep duration were recorded. Sleep latency was characterized as the duration between the drug injection and the cessation of the righting reflex. Sleep duration was characterized as the interval from the sustained absence of the righting reflex for more than 30 s until the reinstatement of the righting reflex.

### 3.6. HE Staining

Brain tissues were fixed for 48 h. Subsequently, they were embedded and sectioned coronally to a thickness of 3 μm. The sections were dried at 45 °C for one hour, followed by deparaffinization and dehydration. The tissues were stained with hematoxylin, differentiated with ethanol, and then thoroughly rinsed. Intensification or restoration of the blue color was achieved by treatment with 0.6% ammonia, followed by another round of rinsing. The tissues were further processed by staining with eosin, dehydrating, and subjecting them to specific treatment to enhance their transparency. The sections were then sealed with neutral gum. Finally, the prepared samples were placed under the microscope, and histomorphology was observed at a magnification of ×200.

### 3.7. ELISA Assay

An appropriate amount of brain tissue was homogenized and centrifuged to isolate the supernatant, following the guidelines provided by the ELISA kit (Jiangsu Enzyme Immunity Industry Co., Ltd., Suzhou, China). Moreover, the OD value was determined utilizing a multifunctional microplate reader at 450 nm. The contents of GABA, 5-HT, and BDNF in the brain tissue of each group of animals were compared. The obtained results underwent analysis through a *t*-test, with a significance level set at *p* < 0.05.

### 3.8. RT-qPCR Assay

Total RNA was obtained from brain tissues by employing the M5 HiPer Universal RNA Mini Kit kit (Polymeric Biotechnology Co., Ltd., Beijing, China). Subsequently, it was reverse-transcribed to synthesize cDNA utilizing the M5 Sprint qPCR RT kit with a gDNA remover kit (Polymeric Biotechnology Co., Ltd., Beijing, China). Moreover, RT-qPCR was performed utilizing the 2X M5 HiPer SYBR Premix EsTaq kit (PolyMirae Biotechnology Co., Ltd., Beijing, China). The 2^−ΔΔCt^ method was employed for relative quantitative analysis. Primer sequences for 5-HT1AR, GABAARα1, BDNF, and GAPDH were synthesized via Sevier Bio Ltd. (Suresnes, France) (Table 2).

### 3.9. Western Blotting Analysis

Total proteins were extracted from brain tissues employing RIPA lysis buffer, and their concentrations were assessed via the BCA protein assay kit (Boster Biological Technology Co., Ltd., Wuhan, China). Samples were then separated on 8–12% SDS-polyacrylamide gels and subsequently transferred onto PVDF membranes. The membranes were blocked in 5% skimmed milk powder for two hours, followed by overnight incubation with specific primary antibodies at 4 °C. The primary antibodies employed were as follows: 5-HT1AR (1:500), (Servicebio, AC231209084), GABAARα1 (1:800), (Servicebio, AC231216033) and BDNF (1:500), (Servicebio, AC231209053). After three washes, the membranes were treated with HRP-anti-conjugated secondary antibodies (1:1000 dilution) for two hours and visualized by an enhanced chemiluminescence kit. The obtained results underwent analysis through a *t*-test, with a significance level set at *p* < 0.05.

### 3.10. Preparation of Control and Test Solution

Control: Appropriate amounts of magnoflorine, spinosin, rutin, 6‴-feruloylspinosin, Jujuboside A1, Jujuboside A, Jujuboside B, betulinic acid, ceanothic acid, and alphitolic acid were precisely weighed. Subsequently, 70% methanol was added to prepare the individual mother solutions with a concentration of 1 mg/mL for each component. Following this, the mother solution of each component of the control was precisely aspirated, and 70% methanol-water was introduced to prepare a mixed working solution of the control. This solution contained 320 μg/mL of magnoflorine, 200 μg/mL of spinosin, 180 μg/mL of 6‴-feruloylspinosin, 200 μg/mL of Jujuboside A, and 100 μg/mL of Jujuboside B.

Test: Precisely 1.00 g of ZSS, ZSF, and ZSKS (through the No. **4** sieve) were added in a 100 mL conical flask. They were then extracted with 20 mL of 70% ethanol-water heated at reflux for two hours and filtered. The filtrate was washed with 5 mL of 70% ethanol water. Subsequently, the washings and filtrates were combined, and the solvent was evaporated until complete removal was achieved, resulting in a dry residue. Upon dissolution with methanol, the sample was transferred to a 10 mL volumetric flask. Moreover, methanol was introduced up to the mark, and the solution was shaken well and filtered. The resulting filtrate constituted the test sample. 

### 3.11. UPLC-QTOF-MS Analysis

UPLC-QTOF-MS experiments were conducted on various components of ZS utilizing an Agilent 1290 high-performance liquid chromatography-tandem with an AB Sciex 5600 + Q-TOF mass spectrometer (Sciex, Framingham, MA, USA). The system configuration was as follows: a chromatographic column ACQUITY UPLCTM HSS T3 (50 mm × 2.1 mm, 1.8 μm); mobile phase constituting of 0.1% formic acid (A)-acetonitrile (B); gradient elution (0–2 min, 10–20% B; 2–8 min, 20% B; 8–15 min, 20–37% B; 15–18 min, 37–40% B; 18–22 min, 40~95% B; 22–26 min, 95% B; 26–27 min, 95~10% B; 27–30 min, 10% B); a volume flow rate of 0.3 mL/min; an injection volume of 2 μL; and column temperature set at 30 °C. The mass spectrometry analysis was conducted in both positive and negative ion modes over a scanning range of 100~2000 *m/z*. High-purity N_2_ served as the auxiliary spray ionization and desolventizing gas, while nitrogen was employed as the atomizer and collision gas. The specific parameters were as follows: atomizer temperature set at 320 °C with atomizer flow rate at 8 L/min; sheath gas temperature was set at 350 °C with sheath gas flow rate at 11 L/min; capillary voltage set at 3500 V for both positive and negative mode; nozzle voltage set at 300 V for positive mode and 1000 V for negative mode; dissociation voltage maintained at 130 V.

### 3.12. Compound Structure Analysis

A database of chemical compounds sourced from various parts of ZS was compiled. A tabular format featuring compound names, molecular weights, molecular formulas, representative fragments, and medicinal parts of the compounds was integrated into Library View to create a database of the chemical components of ZS. The sample data were imported into Master View and matched with the established database for mass spectrometry data. Results exhibiting a high degree of match (compounds highlighted in green within the results list) were exported. The qualitative identification of the chromatographic peaks in ZS was conducted through database matching, comparison with controls, analysis of sample mass spectrometry dissociation fragmentation, comparison with literature data, and assessment of compound structural composition.

## 4. Conclusions

In this investigation, sleep latency and duration were assessed through the establishment of a PCPA mouse model of insomnia. Various components of ZS demonstrated efficacyin ameliorating insomnia symptoms. Notably, ZSS exhibited the most potent sedative effect, followed by ZSF and ZSKS. Subsequently, the chemical composition of different parts of ZS was further examined by employing the UHPLC-LTQ-Orbitrap-MS technique. The shared chemical basis contributing to the sedative and hypnotic potency of different parts of ZS may involve the presence of compounds such as (**1**) magnoflorine, (**8**) betulinic acid, (**9**) ceanothic acid, and (**10**) alphitolic acid. Furthermore, the superior efficacy of ZSS compared to ZSF and ZSKS may be attributed to the presence of compounds such as 6‴-feruloylspinosin, Jujuboside A1, and Jujuboside A, which are not found in ZSF and ZSKS. The superior effectiveness of ZSF over ZSKS may be due to the presence of compounds such as spinosin, rutin, and Jujuboside B, which are found in ZSF but not in ZSKS. It provides a basis for further elucidation of the substance basis responsible for the medicinal effects of ZS and the development and utilization of its resources.

## Figures and Tables

**Table 1:** Mass spectral data of compounds from different parts of ZS isolated by UPLC.

No.	RT (min)	Molecular Formula	Ion Mode	Measured Value	Theoretical Value	Fragment Ion Information	Presumed Compound	Distributed Sites
1	3.735	C_20_H_24_NO_4_	[M+H]^+^	341.1673	341.1699	297.1102 [M+H-(CH_3_)_2_NH]^+^, 282.0872 [M+H-(CH_3_)_3_NH]^+^, 265.0842 [M+H-(CH_3_)_3_NH-OH]^+^, 237.0896 [M+H-(CH_3_)_3_NH-OH-CO]^+^, 222.0661 [M+H-(CH_3_)_4_NH-OH-CO]^+^	Magnoflorine	ZSS ZSF ZSKS
2	4.742	C_28_H_32_O_15_	[M+H]^+^	608.1777	608.1690	489.1125 [M+H-C_4_H_8_O_4_]^+^, 429.1166 [M+H-C_4_H_8_O_4_-C_2_H_4_O_2_]^+^, 351.0856 [M+H-(C_4_H_8_O_4_)_2_-H_2_O]^+^, 327.0852 [M+H-(C_4_H_8_O_4_)_2_-C_2_H_2_O], 297.0749 [M+H-(C_4_H_8_O_4_)_2_-C_2_H_2_O-CH_2_O]^+^	Spinosin	ZSS ZSF
3	4.942	C_27_H_30_O_16_	[M+Na]^+^	633.1382	633.145	463.0971 [M-Rha]^−^, 301.0486 [M-Rha-Glc]^−^	Rutin	ZSS ZSF
4	8.074	C_38_H_40_O_18_	[M+H]^+^	784.225	784.2118	665.1028 [M+H-C_4_H_8_O_4_]^+^, 609.1147 [M+H-C_4_H_8_O_4_-C_2_O_2_]^+^, 429.1163 [M+H-C_4_H_8_O_4_-C_2_O_2_-OGlc]^+^, 351.0842 [M+H-C_4_H_8_O_4_-C_2_O_2_-OGlc-C_2_H_2_O_2_-H_2_O]^+^, 327.0846 [M+H-C_4_H_8_O_4_-C_2_O_2_-OGlc-C_2_H_2_O_2_-H_2_O-C_2_H_2_O]^+^, 177.0541 [M+H-C_4_H_8_O_4_-HCOOH-Glc-C_2_H_2_O_2_-H_2_O-C_2_H_2_O-C_5_H_10_O_5_]^+^	6‴-Feruloylspinosin	ZSS
5	16.957	C_58_H_94_O_26_	[M+Na]^2+^	626.2857	626.2975	1075.5155 [M+Na-Xyl]^+^, 1057.4197 [M+Na-Xyl-H_2_O]^+^, 913.4130 [M+Na-Xyl-Glc]^+^, 751.2672 [M+Na-Xyl-2Glc]^+^, 605.1573 [M+Na-Xyl-2Glc-Rha]^+^, 455.3474 [M+Na-Xyl-2Glc-Rha-Ara]^+^	Jujuboside A_1_	ZSS
6	17.587	C_58_H_94_O_26_	[M+Na]^2+^	626.2857	626.2975	1075.5024 [M+Na-Xyl]^+^, 1057.3259 [M+Na-Xyl-H_2_O]^+^, 913.3430 [M+Na-Xyl-Glc]^+^, 751.3115 [M+Na-Xyl-2Glc]^+^, 605.2128 [M+Na-Xyl-2Glc-Rha]^+^, 455.1573 [M+Na-Xyl-2Glc-Rha-Ara]^+^	Jujuboside A	ZSS
7	18.512	C_52_H_84_O_21_	[M+Na]^2+^	545.2591	545.271	913.4517 [M+Na-Xyl]^+^, 895.1880 [M+Na-Xyl-H_2_O]^+^, 751.2630 [M+Na-Xyl-H2O-Glu]^+^, 605.3415 [M+Na-Xyl-Glc-Glu]^+^	Jujuboside B	ZSS ZSF
8	21.752	C_30_H_48_O_3_	[M+H]^+^	457.1672	457.352	439.1913 [M+H-H_2_O]^+^, 411.1404 [M+H-H_2_O-CO]^+^, 281.2958 [M+H-H_2_O-CO-C_8_H_18_O]^+^, 248.9924 [M+H-H_2_O-CO-C_8_H_18_O-(CH_4_)_2_]^+^, 202.9980 [M+H-H_2_O-CO-C_8_H_10_O-(CH_4_)_2_-C_3_H_10_]^+^, 119.0855 [M+H-H_2_O-CO-C_8_H_10_O-(CH_4_)_2_-C_3_H_10_-C_6_H_12_]^+^	Betulinic acid	ZSS ZSF ZSKS
9	22.35	C_30_H_46_O_5_	[M+H]^+^	487.3376	487.3262	459.0417 [M+H-CO]^+^, 312.3534 [M+H-CO-C_7_H_14_O_3_]^+^, 235.1782 [M+H-CO-C_7_H_14_O_3_-C_5_H_17_]^+^, 190.9958 [M+H-CO-C_7_H_14_O_3_-C_5_H_17_-C_3_H_8_]^+^	Ceanothic acid	ZSS ZSF ZSKS
10	23.416	C_30_H_48_O_4_	[M+Na]^+^	495.3442	495.3469	437.3412 [M+H-CH_2_O-CO]^+^, 338.3424 [M+H-CH_2_O-CO-C_6_H_11_O]^+^	Alphitolic acid	ZSS ZSF ZSKS

**Table 2:** Primer sequences.

Name of Primes	Upstream	Downstream
BDNF	GCCCATGAAAGAAGTAAACGTCC	AGTGTCAGCCAGTGATGTCGTC
5-HT1AR	ACTCCACTTTCGGCGCTTTC	GGCTGACCATTCAGGCTCTTC
GABAARα1	CCAAGTCTCCTTCTGGCTCAAC	CTTTTCTGGAACCACGCTTTTG
GAPDH	CCTCGTCCCGTAGACAAAATG	TGAGGTCAATGAAGGGGTCGT

## Data Availability

Data are contained within the article.

## Abbreviations

diazepam (DZP), γ-aminobutyric acid (GABA), 5-hydroxytryptamine (5-HT), brain-derived neurotrophic factor (BDNF), central nervous system (CNS), GABA A-type receptor (GABAAR), traditional Chinese medicine (TCM), Zizyphus spinosus Hu (ZS), ZS kernel (ZSS), ZS husk (ZSKS), ZS flesh (ZSF), Kunming (KM), specific pathogen-free (SPF).
